# Learning Through Oral Case Presentations and Impact on Medical Students' Clinical Training and Career Development: A Mixed‐Method Study

**DOI:** 10.1111/tct.70242

**Published:** 2025-10-27

**Authors:** Hajime Kasai, Rintaro Imafuku, Kiyoshi Shikino, Hiroshi Tajima, Ikuo Shimizu, Kaho Hayakawa, Chihiro Kawakami, Shoichi Ito, Takuya Saiki

**Affiliations:** ^1^ Research Field of Health Professions Education, Graduate School of Medicine Gifu University Gifu Japan; ^2^ Department of Medical Education, Graduate School of Medicine Chiba University Chiba Japan; ^3^ Health Professional Development Center Chiba University Hospital Chiba Japan; ^4^ Department of Respirology, Graduate School of Medicine Chiba University Chiba Japan; ^5^ Medical Education Development Center Gifu University Gifu Japan; ^6^ Nursing Research Promotion Center, Graduate School of Nursing Nagoya City University Nagoya Japan; ^7^ Department of General Medicine, Graduate School of Medicine Chiba University Chiba Japan; ^8^ Department of Community Oriented Medical Education, Graduate School of Medicine Chiba University Chiba Japan

**Keywords:** academic conference, case presentation, clinical clerkship, medical student

## Abstract

**Introduction:**

While oral case presentations at academic conferences (OCPCs) require lengthy preparation and active involvement, they offer unique learning opportunities for medical students during clinical clerkships (CCs). However, students' specific educational experiences and long‐term impacts remain underexplored. This study clarifies the significance of OCPCs and their influence on CCs, residency and career development.

**Methods:**

A mixed‐methods approach was employed, combining questionnaire data with interviews of physicians who made OCPCs during CCs in the field of internal medicine at Chiba University. The approach focused on learning experience and the impact of OCPCs on CCs, residency and career development. Interview data were analysed using qualitative content analysis and applying a Social Cognitive Career Theory (SCCT) framework.

**Results:**

Twenty‐two physicians responded to the questionnaire survey, and 17 (77.3%) selected internal medicine as their specialty. Thirteen (59.1%) selected the department in which they had experienced OCPCs as their subspecialty area. They perceived their experiences of OCPCs as highly beneficial for CC and residency. Key categories identified through interviews with 10 physicians included enhanced presentation skills, clinical practice and relationships with supervising doctors. OCPCs also influenced career development, as selecting specialties aligned with their OCPCs increased motivation for academic activity and education among junior colleagues.

**Conclusion:**

During CC, OCPCs may enhance presentation skills, support clinical practice and strengthen professional relationships. They can also influence specialty choice, encourage academic and educational engagement and potentially yield long‐term benefits for clinical training, residency preparations and early career development. Additionally, OCPCs may contribute to the development of self‐efficacy, consistent with SCCT.

**Abbreviations**
CC: clinical clerkship
OCPCs: oral case presentations at academic conferences
SCCT: Social Cognitive Career Theory
SD: standard deviation

## 1 Introduction

Academic conferences offer opportunities for participants to present cases as oral presentations, share new findings and network with peers [1, 2]. Oral case presentations at academic conferences (OCPCs) require clinical information and a literature review, summarized in slides or posters. This necessitates further preparation, including gathering detailed case progress, integrating literature and using presentation software. Thus, OCPCs require a broad set of skills.

*This necessitates further preparation, including gathering detailed case progress, integrating literature and using presentation software*.

During clinical clerkships (CCs), case presentations are key learning opportunities for medical students, involving skills such as information organization and oral presentation [3, 4]. These presentations facilitate sharing case details among team members, assisting students in understanding pathophysiology, encouraging them to reflect on clinical reasoning and offering supervisors assessment opportunities [5]. However, students often lack guidance on information to include, and supervisors do not provide adequate support, leading to confusion and pressure to perform well in their presentations [6, 7]. The lack of a clearly defined teaching approach makes it difficult for students to learn effectively, leading them to treat case presentations as a mere formality aimed at avoiding post‐presentation questions [6, 8, 9]. Therefore, explicit guidance and evaluation plans are required for case presentations [8, 10]. Whether medical students encounter similar challenges in presenting cases during OCPCs as they do in CC, or if the learning process differs between the two, remains unclear.

Several studies have explored the educational value of conference participation among medical and undergraduate students. Mabrouk et al. and Amgad et al. emphasize its role in fostering research interest [11, 12], whereas Muhandiramge et al., Barrios‐Anderson et al. and Akhter et al. examine students' experiences and career‐related outcomes such as residency placement [13, 14, 15]. Other studies, including those by Sheu et al., Ma et al. and Liao et al., investigate academic engagement and its influence on career development [16, 17, 18]. While these reports highlight the benefits of early academic involvement, they offer limited insight into how medical students learn through OCPCs, how the educational processes involved and how these experiences relate to future clinical practice and career development.

This study addresses the following research questions (RQs): (1) To what extent do medical students perceive OCPCs during CC as beneficial for their subsequent CC, residency and career development, and are these experiences associated with the career‐related outcomes, such as specialty choice? (2) What do medical students learn through OCPCs during CC? (3) How do these experiences impact their residency and career development? Clarifying these questions will enhance our understanding of the educational value of OCPCs as learning activities during CC and inform future curriculum design by evaluating whether such activities should be intentionally integrated to support students' academic and professional development.

*Clarifying these questions will enhance our understanding of the educational value of OCPCs as learning activities during CC*.

## 2 Methods

### 2.1 Ethical Approval

This study was approved by the Ethics Committee of Gifu University (approval no. 2023‐274). The study database was anonymized.

### 2.2 Study Design

This study employed a mixed‐methods approach, combining quantitative and qualitative data collection and analysis. A follow‐up survey, including questionnaires and interviews, was conducted with graduates to gather insights into their experiences. The qualitative component was grounded in a constructivist paradigm [19], allowing for exploration of participants' experiences and perspectives. The first author, experienced in CC, guided the students in clinical and academic activities. An explanatory sequential design combined questionnaire analysis with interviews. Qualitative content analysis, an inductive method, was applied to analyse the interviews, enabling both qualitative and quantitative insights [20]. Additionally, in this study, the phrase ‘long‐term’ refers to the period approximately 3 to 7 years after graduation, encompassing the latter part of residency training and the early stages of postresidency career development.

We used ChatGPT‐4o (OpenAI) as a language support tool to assist with translating the manuscript from Japanese to English and refining the phrasing. The final content was reviewed and revised by the authors, who take full responsibility for the accuracy and interpretation of the manuscript.

### 2.3 Theoretical Framework

We applied Social Cognitive Career Theory (SCCT) as a theoretical framework for interpreting the impact of OCPCs on students' CCs, residency and career development. Grounded in Bandura's Social Cognitive Theory, SCCT explains how individuals develop career interests and make career‐related decisions through the interplay of self‐efficacy, outcome expectations and personal goals [21, 22]. These constructs influence behaviour and learning and are themselves shaped by four primary factors: mastery experiences, vicarious experiences, verbal persuasion and physiological and affective states [23]. SCCT has been used in previous studies to explore career development in medical education contexts [16, 17, 18]. In our study, we adopted SCCT to clarify how students' experiences with OCPCs may strengthen their self‐efficacy and inform future career choices through these four factors of influence. This framework allowed for a structured interpretation of the learning and motivational effects of OCPCs.

### 2.4 Setting

In Japan, medical education follows a 6‐year curriculum based on the model core curriculum of the Ministry of Education, Culture, Sports, Science and Technology [24]. The first 4 years cover preclinical education, followed by CC. After graduation, students complete a 2‐year residency before choosing a specialty. Additionally, the third to seventh years after graduation typically correspond to the stage following residency, during which physicians engage in training to become board‐certified specialists. This period aligns with fellowship training in North America or postresidency specialty training in other countries.

Effective research presentations, considered a core competency, require summarizing research in a thesis, report or conference presentation [24]; however, methods vary by university. At the target university, the ‘Scholarship’ program assigns medical students to research laboratories, engaging them in activities from the first to third years, culminating in an optional poster presentation. During CC, presentation strategies are guided by students and supervisors and lack concrete curriculum guidelines.

### 2.5 Participants

At the Chiba university, CCs are conducted from the fourth year (November) to sixth year (October), comprising approximately 120 students annually and involving rotations lasting 1–4 weeks. We targeted physicians who participated in CCs in April 2017–November 2021 and indicated interest in internal medicine and academic activities. Participants were those who had presented OCPCs, completed 2 years of clinical residency and were in their third–seventh year postgraduation. The faculty of internal medicine departments helped identify eligible physicians. Invitations were disseminated via email. Participant numbers were based on practical feasibility rather than sample size.

### 2.6 Data Collection

#### 2.6.1 Questionnaire

To address RQ1, a questionnaire, developed from a literature review, examined respondent characteristics and key themes [25] grouped into three areas: OCPC experience; post‐OCPC academic activity (e.g., case and research reports); and the impact of OCPCs on CC, residency and career development. Questions included demographics such as age, sex, years since graduation and specialty—totaling 23 items. RI, KS and IS reviewed and revised the draft with input from five resident physicians. The final questionnaire included 17 items (Table S1).

Three items examined students' self‐evaluation regarding the utility of OCPCs to CC (Q15) and residency (Q16), and their influence on specialty choice/career development (Q17), respectively. Responses were scored on a 5‐point Likert scale ranging from 1 (*Not useful at all* [Q15, 16]; *No influence at all* [Q17]) to 5 (*Very useful* [Q15, 16]; *Very influential* [Q17]).

#### 2.6.2 Semistructured Interviews

To further explore RQ2 and RQ3, semistructured interviews were conducted online via Zoom in May–July 2024 to evaluate the impact of learning on CC, residency and career development. Researchers (HK, KS and HT) with no conflicts of interest, such as employment or supervisory roles, conducted the interviews.

Participants were selected through convenience sampling from those who had completed the questionnaire and consented to participate. Additionally, we included individuals who had rated the impact of their OCPC experience on future career development as either 4 or 5 in the questionnaire. This criterion was intended to ensure rich and relevant responses, especially regarding our main RQ related to career development.

An interview guide based on the RQs was collaboratively developed (Table S2). Participants were asked the following questions: ‘What did you learn from OCPCs and how did you learn it?’ ‘How did this impact your subsequent CC and residency?’ ‘How did it influence your career?’

### 2.7 Data Analysis

Quantitative data were expressed as mean and standard deviation (SD). Audio‐recorded interview data were transcribed verbatim. Qualitative content analysis was applied to each transcript [20], combining descriptions of manifest content with interpretations of latent content. HK and HT independently read and coded the transcripts and then discussed and agreed on the final coding. Inter‐rater reliability, measured by the Kappa coefficient, was 0.69 after code revisions (0.6–0.8 = substantial agreement).

In the secondary analysis, categories and subcategories were developed based on background factors. HK, KS and RI iteratively reviewed and revised the results until consensus was reached.

## 3 Results

### 3.1 Questionnaire Results

Twenty‐two of the 24 physicians (91.7%) participated. Details on participant demographics and subspecialty distribution are presented in Table 1.

**TABLE 1:** Questionnaire survey results (*n* = 22).

Item	Result
Q1. Age (years). mean (SD)	28.8 (2.3)
Q2. Sex. male/female	16/6
Q3. Years after graduation. *n* (%)	
3 years after graduation	6 (27.2)
4 years after graduation	5 (22.7)
5 years after graduation	6 (27.2)
6 years after graduation	3 (13.6)
7 years after graduation	2 (9.1)
Q4. Department. *n* (%)	
Internal medicine	17 (77.3)
Plastic surgery	1 (4.5)
General medicine	1 (4.5)
Paediatrics	1 (4.5)
Psychiatry	1 (4.5)
None	1 (4.5)
Q5. Subspecialty of internal medicine. *n* (%)	
Respiratory medicine	9 (40.1)
Endocrinology and metabolism, diabetic medicine	3 (13.6)
Cardiology	2 (9.1)
Gastroenterology	1 (4.5)
Collagen disease and rheumatology	1 (4.5)
Department of haematology	1 (4.5)
Q6. Time of the first OCPC. *n* (%)	
First half of the 5th year (April–September of the 5th year)	6 (27.3)
Second half of the 5th year (October–March of the 5th year)	3 (13.6)
First half of the 6th year (April–September of the 6th year)	12 (54.5)
Second half of the 6th year (October–March of the 6th year)	1 (4.5)
Q7. Format of the first OCPC. *n* (%)	
Oral presentation (face‐to‐face)	9 (40.9)
Oral presentation (online)	8 (36.4)
Poster presentation (face‐to‐face)	2 (9.1)
Poster presentation (face‐to‐face) and oral presentation (face‐to‐face)	2 (9.1)
Poster presentation (online)	1 (4.5)
Q8. Type of conference where the first OCPC was completed. *n* (%)	
General meetings and annual meetings	12 (54.5)
Local area meetings	10 (45.5)
Q9. The opportunity that led to the first OCPC. *n* (%)	
From the supervising physician	13 (59.1)
From oneself	4 (18.2)
From both sides	5 (22.7)
Q10. Preparation period for the first OCPC (months). mean (SD)	4.1 (2.0)
Q11. Number of OCPC experiences during the CC period (while attending medical school) after the first OCPC. mean (SD)	0.8 (1.2)
3 times. *n* (%)	3 (13.6)
2 times. *n* (%)	3 (13.6)
1 time. *n* (%)	2 (9.1)
No presentation experience. *n* (%)	14 (63.6)
Q12. Experience in writing academic papers during the residency period (while attending medical school) after the first OCPC. *n* (%)	
English original paper.	3 (13.6)
English case report.	4 (18.2)
Japanese original paper.	1 (4.5)
No experience in academic writing.	16 (72.7)
Q13. Number of presentations at academic conferences during the residency (2 years). mean (SD)	1.4 (1.3)
4 times. *n* (%)	2 (9.1)
3 times. *n* (%)	2 (9.1)
2 times. *n* (%)	5 (22.7)
1 time. *n* (%)	7 (31.8)
No presentation experience. *n* (%)	6 (27.3)
Q14. Experience in writing academic papers during the residency period (2 years). *n* (%)	
Original papers in English	1 (4.5)
English case report	3 (13.6)
No experience in academic writing	18 (72.7)
Q15. Usefulness of the experience and learning of OCPC in subsequent CC. mean (SD)	4.2 (0.9)
Q16. Usefulness of the experience and learning of OCPC in postgraduate residency (2 years). mean (SD)	4.3 (0.8)
Q17. Influence of OCPC experience and learning on career choice. mean (SD)	3.6 (1.2)

Abbreviations: CC, clinical clerkship; OCPC, oral case presentation at an academic conference; SD, standard deviation.

Twelve (54.5%) OCPCs occurred in the first half of the sixth year. Thirteen (59.1%) and nine (40.9%) respondents experienced in‐person or online OCPCs, respectively. The predominant reason for making presentations was supervisors' recommendations, cited by 13 respondents (59.1%). The mean preparation period for presentations was 4.1 (SD 2.0) months.

During CC after OCPCs, eight participants (36.4%) presented at other conferences, averaging 0.8 (SD 1.2) presentations, with a maximum of four by two participants. Eight (36.4%) authored case reports and original articles, including works in Japanese and English. Throughout the 2‐year residency, 16 (72.7%) presented at conferences, averaging 1.4 (SD 1.3) presentations (maximum four by two participants). One (4.5%) and three (13.6%) respondents wrote an English‐language article and English‐language case reports, respectively.

**FIGURE 1:**
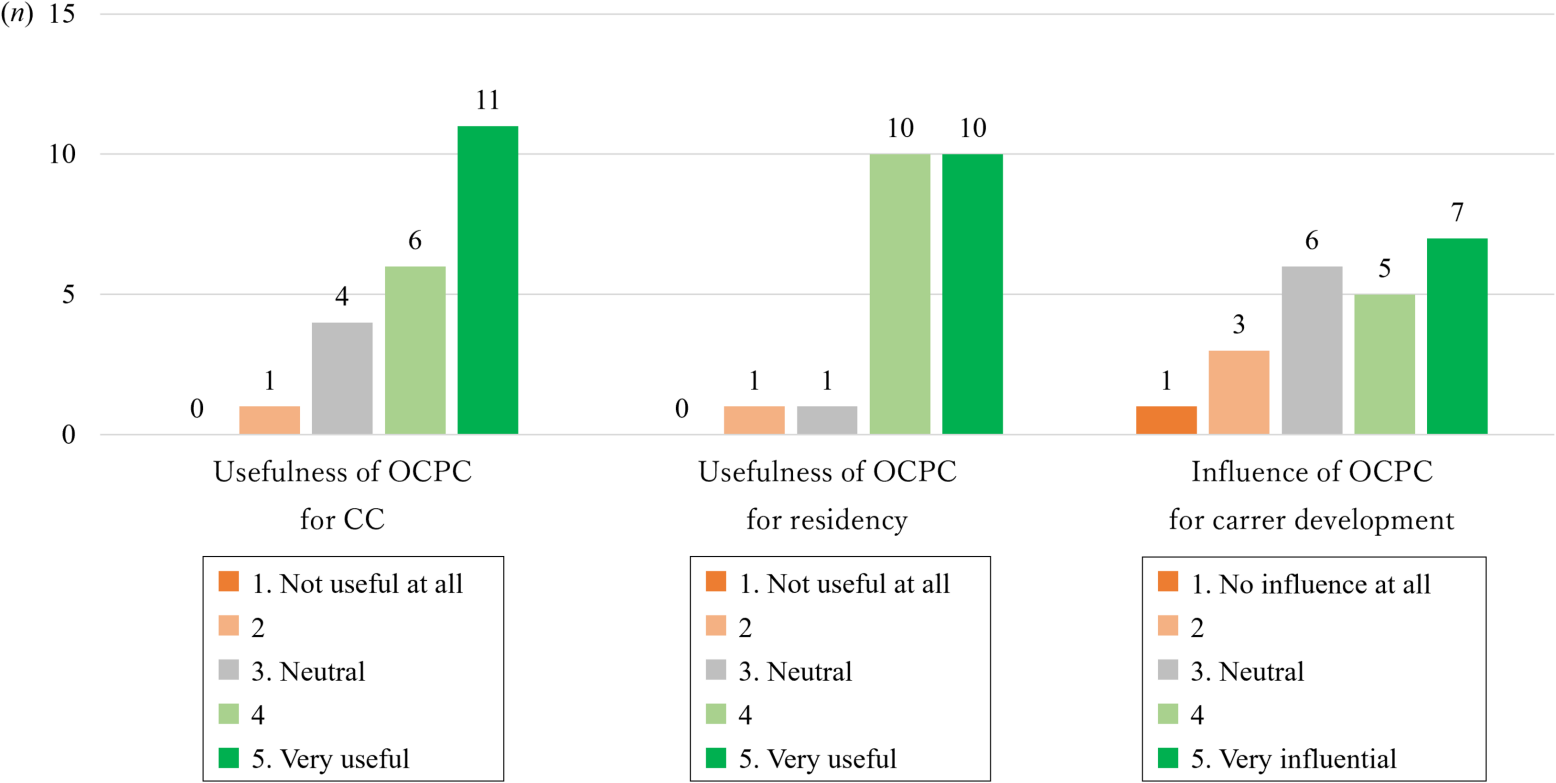
Graph of the impact of OCPCs on subsequent CC, residency and career in questionnaire surveys (*n* = 22). CC, Clinical clerkship; OCPC, Oral case presentation at an academic conference.

The usefulness of learning from OCPCs in subsequent CCs and residency was rated as 4.2 (SD 0.9) and 4.3 (SD 0.8), respectively. As shown in Figure 1, a higher proportion of participants responded that there was greater usefulness during their residency. In contrast, the impact of these experiences on career development was lower, with a mean score of 3.6 (SD 1.2), compared to their impact on CC and residency. However, responses were polarized, with 12 participants (54.5%) selecting ‘5’ or ‘4’ and 10 participants (45.5%) selecting ‘1’ to ‘3’ (Figure 1). Among the 13 participants who chose the same specialty as a department in which they had performed OCPCs, the career impact score was notably higher at 4.0 (SD 0.3) compared to 3.1 (SD 0.4) for those who selected other specialties (*p* = 0.092).

### 3.2 Semistructured Interview Results

In response to the questionnaire, 18 physicians (81.8%) indicated their willingness to participate in semistructured interviews. Of these, 12 had indicated experiencing a strong impact from their OCPCs. From this subgroup, participants were selected using convenience sampling, prioritizing individuals who were accessible and likely to provide rich, reflective insights. The final sample size (10 interviews) was determined based on theoretical saturation, as no new themes emerged thereafter (Table S3). The average interview time was 38 min (SD 10 min).

Altogether, 509 codes were extracted from content analysis, including 92 codes for ‘The learning process of OCPCs’, 154 codes for ‘Lessons learned through OCPCs on CC’, 149 codes for ‘The impact of learning through OCPCs on CC and residency’, and 114 codes for ‘The impact on career development’.

### 3.3 The Learning Process of OCPCs and Self‐Efficacy, and the Lessons Through OCPCs

The methods learned were classified into 13 subcategories and four categories (Table 2) based on the four factors influencing self‐efficacy.

**TABLE 2:** Content analysis results of interviews with 10 participants: Methods of learning for the OCPC (number of codes: 92) and lessons learned from the OCPC (number of codes: 154).

Methods of learning for the OCPC (number of codes: 92)			
**Category**	**Subcategories**	**Quotes**	**ID**
Enactive mastery experience (39)	Challenge approached with ample preparation (10)	*‘As a student, I had way more time to prepare compared to when I became a working professional’*.	A
	Challenging unexplored areas (8)	*‘I feel like I got to gain a lot of experience doing things I had never done before’*.	H
	Collecting multidimensional information beyond textbooks (8)	*‘As students, we usually just study by looking things up in textbooks, right? But the cases I present at academic conferences are rare diseases, and they are not in textbooks. So, it's really unfamiliar’*.	G
	Handling pressure during presentations on the podium (6)	*‘In a conference presentation, I have to assume that there will not be any help, and that was a really valuable experience, I think’*.	H
	In‐depth understanding and exploration of cases (4)	*‘When organizing the presentation, I made sure to dig deep into the information, getting to the point where I knew everything needed to structure the presentation properly’*.	C
	Stepwise preparation process leading up to the presentation (3)	*‘It was really valuable to be able to challenge myself through the whole process of presenting at a conference and to experience how things flow and what comes next’*.	E
Verbal persuasion (33)	Support from supervisors according to the learner's stage of development (18)	*‘They gave me some examples as starting points, and from there I was able to gradually expand and learn in my own way’*.	C
	Discussion with supervising doctor (12)	*‘Compared to clinical clerkship, it was much more interactive and involved two‐way communication’*.	I
	Collaborative work alongside supervising doctor (3)	*‘A big part of it was being able to experience things together while receiving guidance alongside the supervising doctor’*.	E
Physiological and affective states (11)	Self‐directed inquiry (8)	*‘Since there's a big focus on being proactive, I think the difference in how much you learn sticks with you, and that's probably where the biggest gap is’*.	B
	Gaining a sense of achievement and self‐confidence (3)	*‘I'm really glad I did it. I did not think anything other than that—it really boosted my confidence’*.	H
Vicarious experience (9)	Modelling from presentations by accomplished supervisors or experienced presenters (5)	*‘Since I did not have a foundation at first, I just copied others and gradually figured out, “Oh, this is how it's done”’*.	D
	Inspiration from other participants' presentations (4)	*‘Just listening to the presentations sparked my interest. I found myself thinking, “I want to look into this more”’*.	G
**Lessons learned from the OCPC (number of codes: 154)**			
**Category**	**Subcategories**	**Quotes**	**ID**
Broader information gathering and organization (60)	Utilization of information tailored to the objective (25)	*‘After all, when making slides, I think about what kind of information to cut out, how to make it easy to understand, and how to narrow down the theme of the presentation’*.	D
	Diverse literature search and comprehension based on the objective (24)	*‘After all, I did some research on my own, and I learned to look up various related papers and learn to pull them off, or something like that’*.	F
	Identifying key points of the case (5)	*‘In order to take it to such a place, I think it was probably an opportunity for me to grasp the fact that this is the best place to go’*.	I
	Gathering information beyond the textbook (3)	*‘I thought that maybe I learned a lot of things that I could not understand just by looking at the textbook’*.	B
	Detailed medical history taking with the presentation in mind (3)	*‘I think I also learned how to take a person's medical history in detail before making a presentation’*.	B
Comprehensive preparation and material creation for presentations (48)	The process and importance of preparing presentations (17)	*‘At first, I was taught the whole thing, and then I actually tried it myself, and I got the whole picture to some extent, and then I think it was good for the first time’*.	J
	Creating clear presentation slides, posters and figures (13)	*‘In terms of making slides, I think I learned a lot about graphics, or even when making a table that you can see with a picture from the eye, the size of the letters, the ease of seeing, the sense of unity, the unity of colors, and so on’*.	F
	Methods for oral presentation and preparing for questions (12)	*‘He taught me how to speak this way, so I took a pause, and so on’*.	A
	Structure of OCPC (6)	*‘After all, I was learning how to make a proper PowerPoint and how to structure a presentation’*.	H
New approaches to engaging with cases (32)	In‐depth case analysis (18)	*‘I think I learned a lot about assembling what this case was like’*.	F
	Formulating clinical questions (4)	*‘I do not know how much I know and how much I do not know’*.	D
	Identifying the novelty of the case (4)	*‘I think it was an opportunity to witness something that I thought had some kind of novelty or specificity’*.	I
	Gaining diverse perspectives for the case (4)	*‘I get a lot of opinions, and by getting new perspectives from other teachers, I can broaden my perspective on them’*.	C
	Handling rare diseases and atypical cases (2)	*‘I think it was an opportunity for me to become aware that illness does not come by looking like a textbook’*.	I
The significance and role of academic conferences and academic activities (14)	The educational role of academic conferences (5)	*‘There were a lot of things that I was able to learn about the educational institutions of the academic society’*.	I
	Various research themes presented at academic conferences (5)	*‘It was the first time I participated in the seminar and I saw other presentations, but I was able to understand what kind of things everyone was interested in, what clinicians were passionate about, and how they were interested in their treatment’*.	A
	The significance of case presentations and case reports (4)	*‘Maybe it will be useful to others, and if you get into trouble with it, you may end up somehow’*.	I

Abbreviations: CC, clinical clerkship; OCPC, oral case presentation at an academic conference.

The learning process was analysed using four domains based on self‐efficacy theory (Table 2). Students described how successfully preparing for and delivering OCPCs boosted their confidence, aligning with an enactive mastery experience. They also benefited from developmental feedback (verbal persuasion), emotional triggers for self‐directed learning (physiological and affective states) and observing experienced presenters (vicarious experience). These mechanisms illustrate how OCPCs contributed to building self‐efficacy and active engagement in academic practice based on SCCT.

*Students described how successfully preparing for and delivering OCPCs boosted their confidence, aligning with an enactive mastery experience*.

The lessons learnt were classified into 17 subcategories and four categories (Table 2). These themes reflect how OCPCs fostered technical and cognitive skills (e.g., data organization and presentation design) and promoted a deeper engagement with clinical reasoning and academic professionalism.

### 3.4 The Usefulness of OCPCs in CC and Residency

The usefulness of OCPCs in CC and residency was classified into 19 subcategories and six categories (Table 3). These themes highlight how OCPCs bridged academic and clinical learning by increasing motivation, enhancing information management and strengthening communication skills, all of which were reported as being beneficial during later clinical training.

**TABLE 3:** Content analysis results of interviews with 10 participants: Usefulness of experience of the OCPC in CC and residency (number of codes: 149).

Category	Subcategories	Quotes	ID
Increased motivation for academic activities (44)	Practice in treating patients with an awareness of OCPC (15)	*‘When preparing for a presentation, I realized that I needed to gather information on parts I did not initially think were necessary. I felt like I had to be much more thorough than in regular clinical practice’*.	B
	Increased motivation for OCPC (12)	*‘Seeing cases like this being presented made me think, “I have cases too, and I'd like to present them as well”’*.	G
	Attitude toward learning from others' presentations (9)	*‘I think I started to see things earlier on, like how to design my presentation, or how saying something a certain way could make it clearer. I also began to appreciate the quality of others' presentations’*.	D
	Reduced barriers to OCPC (7)	*‘I think I've become less nervous now, maybe I've gained a bit more confidence’*.	H
	Realization of the enjoyment in academic analysis (1)	*‘It feels like I'm diving deeper into the academic side of things, and it's fun—really fun. Everyone seems to be enjoying it in their own way, and I found that really engaging’*.	D
Improvement in presentation skills (37)	Presentations with a focus on clear communication (22)	*‘I kind of started to understand what the senior doctors wanted to know, and I focused my presentation around that’*.	B
	Increased efficiency in creating presentation materials (15)	*‘Having a solid foundation allowed me to expand from there and build on it’*.	F
Enhancement of information gathering and utilization skills (28)	Enhanced enthusiasm for literature search and comprehension (20)	*‘I think I now see literature as one of the tools for clinical practice, especially when encountering rare diseases. It's something I can turn to as a resource’*.	H
	Approach to solving clinical questions (3)	*‘I feel like I've developed a habit of thinking things through when I encounter something I do not understand’*.	E
	Ability to judge the importance of information (3)	*‘Understanding which information is essential and which is not changes a bit over time. It was difficult as a student, but I felt like it became useful later during my residency’*.	D
	Attitude toward collecting broad clinical information (2)	*‘In everyday clinical practice, I've started to look at the records of various departments’*.	H
Improvement in clinical skills (21)	Improvement in the quality of clinical consideration (9)	*‘I think I've been able to confront my disease from a deeper perspective’*.	C
	Building a foundation in clinical reasoning (6)	*‘I think the perspectives I gained during that time have really helped me build clinical reasoning’*.	C
	Handling rare diseases and atypical cases (6)	*‘I've developed a habit of separating out the typical parts of a case from the atypical ones, especially when something seems a bit unusual’*.	I
Changes in the relationship with supervising doctors (10)	Active discussion with supervising doctors (8)	*‘I do not hesitate to ask questions in discussions anymore. If I'm stuck, I just ask’*.	G
	Establishing an easy consultation relationship with supervising doctors (2)	*‘I feel like I've built a better relationship with my supervisors—it's become easier to consult with them’*.	B
Increased learning motivation (9)	Increased motivation to study clinical medicine (6)	*‘Since there's something to learn from each and every patient, I naturally developed the mindset of wanting to learn as much as I can. I think that was really beneficial’*.	J
	Attitude of connecting learning with personal interests (2)	*‘I've started to tie what I'm interested in now back to what I learned as a student’*.	F
	Challenging clinical internships abroad during the CC (1)	*‘As a study abroad destination at Chiba University, I'm particularly interested in learning more about the field of respiratory transplants’*.	F

Abbreviations: CC, clinical clerkship; OCPC, oral case presentation at an academic conference.

### 3.5 The Impact of Learning Through OCPCs on Career Development

The impact of OCPCs on career development based on SCCT was classified into 14 subcategories and five categories (Table 4). These results suggest that OCPCs influenced students' career trajectories by enhancing interest and self‐efficacy, shaping career goals, guiding concrete choices (e.g., department selection) and refining outcome expectations related to academic and clinical futures.

**TABLE 4:** Content analysis results of interviews with 10 participants: Impact of the OCPC on career development (number of codes: 114).

Category	Subcategories	Quotes	ID
Impact of multifaceted interests (51)	Increased understanding and interest in the respective medical specialty (30)	*‘I realized that internal medicine, where you really have to think things through, is actually pretty interesting. I also found out that there are specialties where you can look after patients while considering different possibilities, even if things aren't entirely clear’*.	I
	Increased interest in research (16)	*‘I came to the conclusion that having some academic experience is definitely beneficial for a doctor’*.	D
	Improved understanding of education (5)	*‘Because I had the experience of being mentored, when it was my turn to teach, I did not see it as much of a challenge’*.	C
Impact on self‐efficacy (22)	Desire to pass on the education received to junior doctors (11)	*‘I felt like I was handed the baton, and now I really want to pass it on to someone else’*.	H
	Proactive attitude towards OCPC (8)	*‘My willingness to dive into things like oral presentations was probably influenced by the experiences I had during that time’*.	C
	Understanding the learning benefits of OCPC (3)	*‘Looking back, I think giving younger people opportunities to have these experiences is the best form of education’*.	D
Impact on choice goals (16)	Trigger for deciding on the respective medical department (9)	*‘I think I was able to dive in because I had already gained some knowledge in the field. If I had known nothing at all, I would not have been able to take the plunge’*.	G
	Maintenance of long‐term professional relationships with supervisors (4)	*‘I think it was really beneficial to have long‐term interactions, not just short‐term exchanges’*.	J
	Motivation for joining own home university (3)	*‘I realized how important it is to be in a department where you feel like you are needed’*.	H
Choice action (14)	Continuous participation in academic conferences (5)	*‘In a way, I am no longer compelled to contemplate conferences so inflexibly, as I comprehend that they resemble a celebration in this regard’*.	E
	Practical relations with doctors of the relevant medical department (5)	*‘I had the opportunity to talk a lot with the doctors in the relevant department and the senior doctors’*.	J
	CC and residency linked to the respective department field (4)	*‘Being able to connect things in different ways and not just glossing over them was really valuable, I think’*.	F
Impact on outcome expectations (11)	Clarification of future career goals (7)	*‘It was great to actually join this department and get a clear sense of how the work flows and who I'd be working with’*.	E
	Awareness of career diversity (4)	*‘I realized that there are so many different paths in life and that doctors with medical licenses follow a wide variety of career trajectories. It was eye‐opening to see that firsthand’*.	I

Abbreviations: CC, clinical clerkship; OCPC, oral case presentation at an academic conference.

## 4 Discussion

**FIGURE 2:**
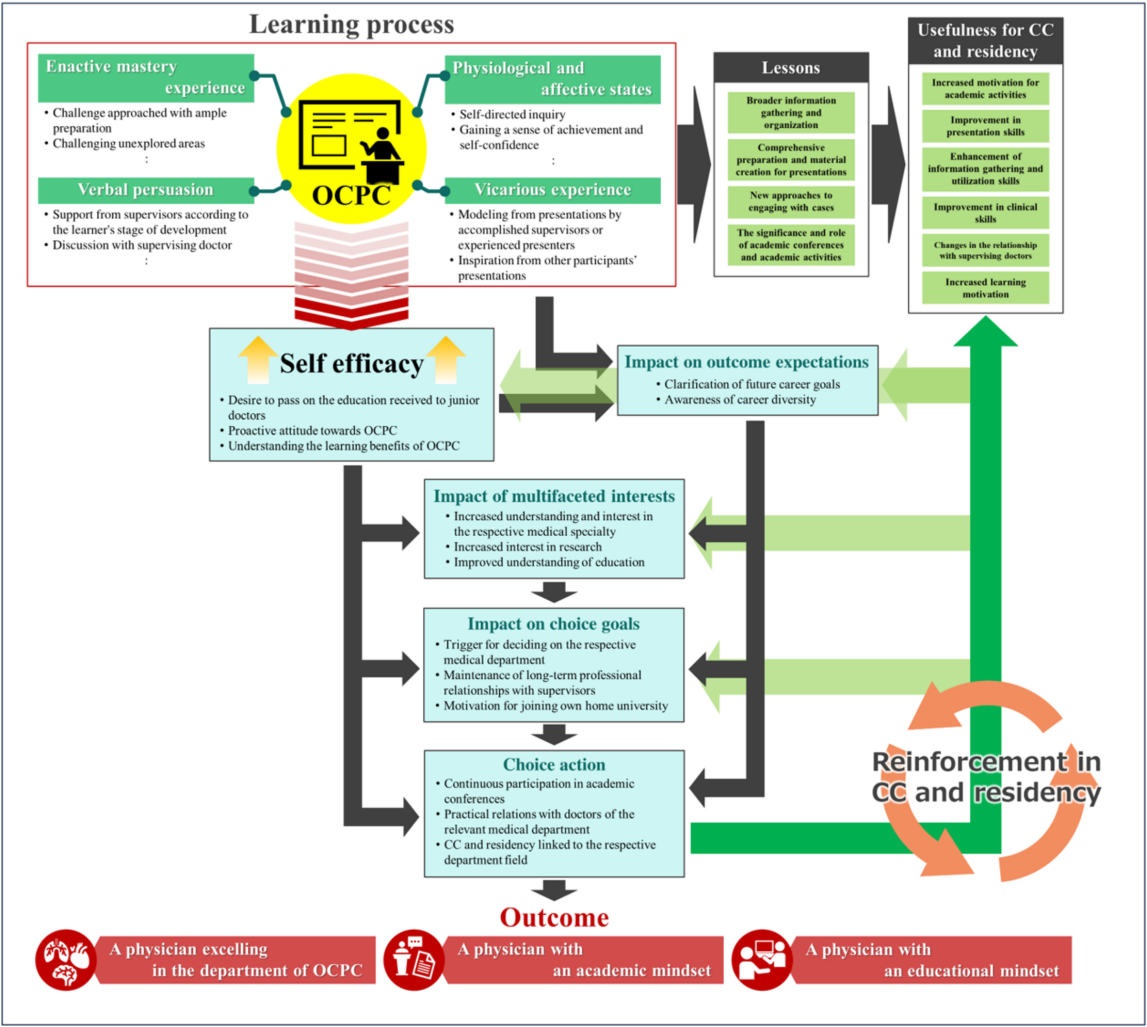
The learning process from OCPCs and their impact on clinical clerkship, residency, and career development based on the framework of Social Cognitive Career Theory. OCPC, Oral case presentation at an academic conference.

To our knowledge, this is one of the few studies to explore what medical students learn through their participation in OCPCs, how these learning experiences unfold and how they influence subsequent CC, residency training and early career development. The findings suggest that long‐term preparation for OCPCs, supported by supervising physicians, enhances presentation abilities and CC competencies while also boosting self‐efficacy. These improvements were reinforced during CC and residency, leading to behavioural modifications. Furthermore, OCPCs fostered relationships with clinical departments, creating a sense of accomplishment that encouraged research engagement, specialty selection and continuous career progression according to SCCT principles. The learning outcomes and impacts of these OCPCs are summarized in Figure 2.

*Long‐term preparation for OCPCs, supported by supervising physicians, enhances presentation abilities and CC competencies while also boosting self‐efficacy*.

For medical students, an OCPC is a challenging yet valuable learning goal that involves early participation in academic conferences. Unlike typical CC experiences, it requires extensive preparation and intensive guidance from supervising doctors. However, short clinical rotations in CC limit the effectiveness of such support [26, 27]. In a report focusing on students who presented their research at conferences, conference presentations were found to offer a less stressful environment for practice and feedback [14]. In our study, preparation time averaged 4 months, allowing repeated explicit instructions from and frequent engagement with supervisors. Conference participation also provided vicarious experiences and promoted metacognition and reflection, which improved self‐efficacy. Thus, OCPCs serve as an effective and efficient learning opportunity.

*OCPCs serve as an effective and efficient learning opportunity*.

The questionnaire results showed high perceived usefulness of OCPCs, with scores of 4.2 (SD 0.9) for CC and 4.3 (SD 0.8) for residency. Regarding applicability, 17 (77.3%) and 20 (90.9%) respondents rated it as useful for CC and residency, respectively. Lessons included academic aspects such as *Comprehensive Preparation for Presentations* and *The Significance of Academic Conferences*, and practical clinical skills such as *Broader Information Gathering* and *New Approaches to Engaging with Cases*. In CC and residency, OCPC learning contributed to *Increased Motivation for Academic Activities*, *Improved Presentation Skills*, *Enhancement of Information Gathering and Utilization Skills* and *Improvement in Clinical Skills*; the benefits thereof extended to medical interviews, physical examinations and evidence‐based medicine applications. While previous studies focus on the career‐related benefits of academic presentations, such as fostering research interest and influencing specialty choice [11, 12, 13, 14, 15, 16, 17, 18], few examine the learning processes involved. Our findings provide new insights into how OCPCs contribute to students' development through repeated feedback, reflective practice and faculty interaction—components aligned with the extant literature on feedback culture [27], learner agency in the workplace [26], structured presentation training [28] and situated participation in clinical communities [29].

*Our findings provide new insights into how OCPCs contribute to students' development*.

The OCPC experience can influence specialty choice and affiliation with university hospital departments. Among the 22 participants, 17 (77.3%) chose internal medicine, and 13 (59.1%) selected the department in which they had presented. Initially interested in internal medicine, these students likely sustained or deepened their interest. The interviews revealed that career influences extend beyond SCCT factors (personal goals, self‐efficacy and outcome expectations), shaping their interests and choices. The experiences varied, with students presenting voluntarily or with supervisors' recommendations, achieving milestones through structured guidance. Through structured guidance and repeated presentation experiences, students achieved success within a professional community, enhancing their self‐efficacy and sense of accomplishment—key components in SCCT [21]. OCPCs offered opportunities for enactive mastery, vicarious learning from peers and positive affective responses supported by supervisors, all of which helped shape students' outcome expectations and broaden their awareness of potential career paths. These experiences further deepened intrinsic motivation to pursue academic engagement, participate in future conferences, collaborate with clinical departments and make informed choices regarding rotations and specialty selection. Recognizing the educational value of conference presentations reduced psychological barriers and sustained students' academic interest. Such developmental processes reflect legitimate peripheral participation in professional communities [29], where students progress from peripheral observers to active contributors. Consistent with findings from Sheu et al. [17], who explored clinical electives using SCCT, OCPCs were found to support career development aligned with students' interests, goals and motivation.

*The OCPC experience can influence specialty choice and affiliation with university hospital departments*.

In the qualitative content analysis, a subcategory emerged in which participants expressed a strong desire to ‘pass on’ the guidance they had received to their juniors, suggesting that OCPCs may foster a sense of educational responsibility and teaching motivation. This aligns with previous studies indicating that early teaching experiences, positive role models and recognition of the value of education contribute to physicians' motivation to teach [30, 31], despite the limited availability of formal training programs. Through OCPCs, students gained a deeper appreciation of educational processes and were inspired to support the learning of junior peers—an experience that may help cultivate future medical tutors and contribute to a sustainable cycle of clinical education [18].

*OCPCs may foster a sense of educational responsibility and teaching motivation*.

This study has several limitations. First, it involved only 22 graduates from one Japanese medical school, potentially introducing bias regarding the sample size, geography and curriculum. Therefore, the findings may lack generalizability to a broader population. Second, focusing on internal medicine specialists may limit the relevance of the results to other disciplines. Third, a time gap of up to 7 years between the case presentations and the study could have introduced recall bias. Fourth, the exploratory design reflects incomplete validation and reliability of the questionnaire and interview guide. Furthermore, external factors (e.g., clinical rotations and residency programs) might have influenced the results. Future studies should use control groups to improve comparison validity.

## 5 Conclusions

OCPCs during CC offer a distinct and effective educational experience that extends beyond standard clerkship activities. Through intensive preparation and close faculty guidance, students enhanced their presentation skills, deepened clinical engagement and cultivated academic interest. These experiences contributed to increased self‐efficacy and supported long‐term career development—including specialty choice and teaching aspirations—aligning with the principles of SCCT. Therefore, OCPCs may function as a catalyst for clinical learning and a foundation for sustained academic and professional growth.

*OCPCs may function as a catalyst for clinical learning and a foundation for sustained academic and professional growth*.

## Author Contributions

**Hajime Kasai:** conceptualization, methodology, investigation, data curation, formal analysis, writing – original draft, writing – review and editing. **Rintaro Imafuku:** conceptualization, methodology, formal analysis, writing – review and editing. **Kiyoshi Shikino:** investigation, data curation, formal analysis, writing – review and editing. **Hiroshi Tajima:** investigation, data curation, formal analysis, writing – review and editing. **Ikuo Shimizu**: methodology, formal analysis, writing – review and editing. **Kaho Hayakawa:** conceptualization, methodology, writing – review and editing. **Chihiro Kawakami:** conceptualization, methodology, writing – review and editing. **Shoichi Ito:** conceptualization, methodology, writing – review and editing, supervision. **Takuya Saiki:** conceptualization, methodology, formal analysis, writing – review and editing, supervision.

## Ethics Statement

This study was approved by the Ethics Committee of Gifu University (approval no. 2023‐274). Informed consent was obtained from the participants before the survey, which was documented online. All methods were performed in accordance with the relevant guidelines and regulations.

## Consent

The authors have nothing to report.

## Conflicts of Interest

The authors declare no conflicts of interest.

## Supporting information


**Table S1:** Questionnaire content, answer format and options.
**Table S2:** Interview guidelines.
**Table S3:** Characteristics of participants who responded to the semistructured interviews (*n* = 10).

## Data Availability

The datasets generated and/or analysed in the current study are available from the corresponding author upon reasonable request.
