# Supplementary material for: Learning Through Oral Case Presentations and Impact on Medical Students' Clinical Training and Career Development: A Mixed‐Method Study
Source: Clin Teach. 2025 Oct 27;22(6):e70242. doi: 10.1111/tct.70242 (PMC12559780; doi:10.1111/tct.70242)
Supplement: Supplementary file 1 — Table S1: Questionnaire content, answer format and options. Table S2: Interview guidelines. Table S3: Characteristics of participants who responded to the semistructured interviews (n = 10). [file TCT-22-e70242-s001.docx]

**Supplementary Table 1.** **Questionnaire content, answer format, and options.**

| Items | Answer (choice) |
| --- | --- |
| 1. Age | Enter a number. |
| 1. Gender | Select from the following options:  Male, female, not answering |
| 1. Years after graduation | Choose from 5 options for 3–7 years after graduation |
| 1. Department | Select from the following options:  Internal Medicine, Pediatrics, Dermatology, Psychiatry, Surgery, Orthopedics, Obstetrics and Gynecology, Ophthalmology, Otolaryngology, Urology, Neurosurgery, Radiology, Anesthesiology, Pathology, Clinical Laboratory, Emergency, Plastic Surgery, Rehabilitation, General Medicine |
| 1. Subspecialty | Select from the following options:  No subspecialty, Gastroenterology, Cardiovascular Medicine, Respiratory Medicine, Hematology, Endocrinology and Metabolism, Diabetes Medicine, Neurology, Nephrology, Collagen Disease, Rheumatology, Gastroenterological Surgery, Thoracic Surgery, Cardiovascular Surgery, Pediatric Surgery, Breast Surgery, Diagnostic Radiology, Radiation Therapy |
| 1. When is the time for the first OCPC? | Select from the following options:  The second half of the 4th year (at the start of the 4th year clinical training to March), the first half of the 5th year (April**–**September of the 5th year), the second half of the 5th year (October**–**March of the 5th year), the first half of the 6th year (April**–**September of the 6th year), and the second half of the 6th year (October**–**March of the 6th year) |
| 1. What was the format of the OCPC? | Oral presentation, poster presentation |
| 1. What type of academic conference did you give your first OCPC? | Select from the following options:  Local meetings, general meetings |
| 1. Was it your own request to give your first OCPC? Is it because you were recommended by your supervisor? | Select from the following options:  Wishing on your own, recommended by your supervisor, both. |
| 1. How long did you prepare for your first OCPC? | Select from the following options  Less than 1 month, 1 month, 2 months, 3 months, 4 months, 5 months, 6 months, 7 months, 8 months, 9 months, 10 months, 11 months, 12 months or more |
| 1. How many OCPC do you give at academic conferences during the CC period? | Select the number of times from the number. |
| 1. Do you have any experience writing academic papers during the CC period (while attending medical school) after your first OCPC? | Select from the following options (multiple answers possible):  No experience in writing academic papers, Japanese case reports, English case reports, Japanese original papers, and English original papers |
| 1. How many OCPC did you give during your residency? | Select the number of times from the number |
| 1. Do you have experience writing academic papers during residency? | Select from the following options (multiple answers possible):  No experience in writing academic papers, Japanese case report, English case report, Japanese original article, English original article |
| 1. To what extent did your experience and learning from OCPC prove useful in your subsequent CC? | 5-point Likert scale  (1: Not useful at all — 3: Neutral — 5: Very useful). |
| 1. To what extent did your experience and learning from OCPC prove useful during your residency? | 5-point Likert scale  (1: Not useful at all — 3: Neutral — 5: Very useful). |
| 1. To what extent did your experience and learning from OCPC influence your career path? | 5-point Likert scale  (1: No influence at all — 3: Neutral — 5: Very influential). |

Note: CC, clinical clerkship; OCPC, oral case presentation at an academic conference.

**Supplementary Table 2.** **Interview guidelines.**

| 1. **Introductory conversation** | Thanking the participants  Statement of the aim of the study  Procuring informed consent  Obtaining permission for recording and filming |
| --- | --- |
| 1. **Flow of the interview: Statements and questions** | 1. Please introduce yourself. 2. Please describe what you learned from your OCPC experience and how you acquired that learning. 3. Please explain how what you learned from your OCPC experience influenced your subsequent CC and residency. 4. If your OCPC experience influenced your career path, please describe it in detail. |
| 1. **Conclusion** | Is there anything else you would like to add on this topic? |

Note: CC, clinical clerkship; OCPC, oral case presentation at an academic conference.

**Supplementary Table 3. Characteristics of participants who responded to the semi-structured interviews (n = 10).**

| No | Years  post-graduation | Sex | Department |
| --- | --- | --- | --- |
| 1 | 4―5 | male | Allergy and Rheumatology |
| 2 | 4―5 | male | Cardiology |
| 3 | 4―5 | male | Diabetes, Metabolism, and Endocrinology |
| 4 | 4―5 | female | Diabetes, Metabolism, and Endocrinology |
| 5 | 4―5 | female | Hematology |
| 6 | 4―5 | male | Respiratory medicine |
| 7 | 4―5 | male | Respiratory medicine |
| 8 | 6―7 | male | Respiratory medicine |
| 9 | 6―7 | male | Respiratory medicine |
| 10 | 6―7 | male | Respiratory medicine |
